# An exploratory study of relationships between the executive functions and spatiotemporal control in a complex motor task: the spatial tapping task

**DOI:** 10.3389/fnhum.2026.1881291

**Published:** 2026-10-19

**Authors:** Mariama Dione, Gaëlle Alhaddad, Marieke Longcamp, Yvonne Delevoye-Turrell

**Affiliations:** 1 CRPN - Centre de Recherche en Psychologie et Neurosciences, UMR 7077, CNRS, Aix-Marseille Université, Marseille, France; 2 SCALab - Sciences Cognitives et Sciences Affectives, UMR 9193, CNRS, Université de Lille, Lille, France; 3 Institut Universitaire de France (IUF), Paris, France

**Keywords:** aging, dementia, cognitive flexibility, monitoring, replication, schizophrenia, shifting, cognitive stability

## Abstract

Executive functions (EFs) are cognitive processes enabling planning, control, and regulation of behavior. A variety of tasks have been developed to evaluate EFs, from complex reasoning to simpler inhibition tasks, reflecting the *diversity* of symptoms observed in patients with executive deficits. Recent research has emphasized their *unity*, proposing three core functions, working memory, inhibition, and switching, that share resources and combine to enable complex tasks. Yet, no single task captures both the unity and diversity of EFs, limiting the development of a unified framework and a balanced assessment. To address this gap, we designed the spatial tapping task, a timing paradigm combining finger tapping and circle drawing. Finger tapping requires working memory for explicit timing control, whereas circle drawing requires anticipatory control akin to switching to control movement trajectory, enabling implicit timing control. Participants produce taps along a circular trajectory with the tempo increasing progressively. A slow tempo favors discrete movements with long pauses between taps, whereas a fast tempo promotes continuous motion. Previous analyses supported a change in timing control mode with increasing tempo. Here, we explored the hypothesis that EFs are engaged accordingly: working memory in the slow/discrete phase, switching in the fast/continuous phase, and inhibition in the intermediate phase, where demands for continuous motion increase. In two studies combined into a single dataset, we compared scores with those from neuropsychological tests using correlations. We found that timing accuracy in the slow/discrete phase was associated with working memory, spatial accuracy in the fast/continuous phase with switching, and longer contact time in the intermediate phase with inhibition. We also explored whether performance was consistent with the EF deficits described in the literature, and the potential contribution of motor output, reporting scores obtained across populations with executive deficits (aging and dementia), combined executive and motor impairments (schizophrenia), and motor expertise (biscriptual writers), along with matched controls. Findings were consistent, although often more specific, and highlighted an important contribution of motor output. Altogether, our results provide initial evidence that the task can be used to investigate EFs, although cognitive, motor, and sensory contributions need to be disentangled to improve its specificity.

## 1 Introduction

Executive functions (EFs) are higher-order cognitive processes that enable the planning, control, and regulation of behavior. Historically defined in neuropsychology, they primarily describe the symptomatology observed in patients with frontal lobe damage (Damasio, 1994; Harlow, 1868; Shallice and Burgess, 1991). This includes changes in personality characterized by increased impulsivity, difficulties in achieving long-term goals, and impaired reasoning. Early research introduced a range of theoretical concepts such as planning, reasoning, impulsivity, and inhibition, alongside tasks designed to assess them (e.g., the Tower of London, Shallice, 1982; or the Wisconsin Card Sorting Test, Grant and Berg, 1948), emphasizing the diversity of symptoms observed in frontal patients. More recent work has highlighted the unity underlying the diversity of EFs (Friedman and Miyake, 2017; Miyake et al., 2000; Miyake and Friedman, 2012) by identifying and distinguishing three core functions, namely working memory, inhibition, and switching, and demonstrating their contribution to more complex tasks using a latent-variable approach (Miyake et al., 2000). This approach has also highlighted the variability among tasks used to evaluate EFs, which may differ in their task demands, reducing comparability even when they are assumed to target the same EF (Burgess et al., 1998; Miyake et al., 2000; Miyake and Friedman, 2012). This highlights the current need to develop novel tasks in which core EFs are coherently organized within a single context and integrated into more complex functions, such as planning (Diamond, 2013; Koziol and Lutz, 2013).

Our aim in the present study is to propose a unifying paradigm for studying EFs and the relationships among them within the context of motor control. Our approach is grounded in the idea that EFs primarily arise from the need to organize complex motor behavior (see Koziol and Lutz, 2013, for a similar perspective). Complex motor tasks therefore provide an ideal context for assessing these functions. Evidence from the motor control literature indicates that motor tasks requiring explicit timing control recruit working memory mechanisms (Bååth et al., 2016; Broadway and Engle, 2011a, 2011b; Brown, 2006; Holm et al., 2017; Maes et al., 2015; Ogden et al., 2011). Conversely, motor tasks requiring the coordination of distinct motor patterns into smooth spatial trajectories, such as fluent fingerspelling (Jerde et al., 2003), skilled piano playing (Winges et al., 2013), or alternating wrist flexion and extension under laboratory conditions (Roerdink et al., 2008, 2012), engage anticipatory mechanisms similar to those underlying task switching (Rogers and Monsell, 1995).

To our knowledge, the relationships between task-switching and this type of spatial task have not yet been investigated. However, both may involve a common principle: minimizing transition costs through anticipatory control associated with distinct task instructions in classic task-switching paradigms (Rogers and Monsell, 1995) and with successive motor commands in spatial tasks (Roerdink et al., 2008). Under laboratory conditions, two anticipatory strategies have been identified: a visual strategy, whereby gaze is oriented toward the transition point between movement patterns, and a musculoskeletal strategy, whereby the upcoming movement is prepared in advance (here, by flexing or extending the wrist; Roerdink et al., 2008). While the former strategy reduces spatial error at the transition, the latter reduces both spatial and temporal errors at this point. Similar strategies may be used in fluent fingerspelling, resulting in the next letter being embedded within the current gesture (Jerde et al., 2003).

The spatial tapping task can be viewed as a combination of finger tapping and circle drawing paradigms studied in motor timing research (Dione and Delevoye-Turrell, 2015; Robertson et al., 1999). During finger tapping, participants are instructed to repeat finger taps according to a specific timing goal, which can be either internally generated or externally provided, for example, by a metronome. The fundamental timing mechanism involves comparing the produced interval with a reference interval in working memory (Creelman, 1962; Gibbon et al., 1984; Treisman, 1963), allowing corrections of subsequent taps when needed (Vorberg and Wing, 1996; Wing and Kristofferson, 1973a, 1973b). This error-based correction mechanism therefore involves monitoring and updating of working-memory representations to adjust subsequent responses (Miyake et al., 2000). This form of explicit timing control is particularly important for producing long intervals (>0.5 s; Dione and Delevoye-Turrell, 2015; Huys et al., 2008, 2010; Repp, 2008). Consistent with this finding, interference paradigms have shown a stronger contribution of working memory during the production of long than short intervals (Bååth et al., 2016; Maes et al., 2015).

During circle drawing, continuous drawing movements are repeated on a sheet of paper displaying a printed circle, most often using a pen, and in accordance with a temporal goal set by a metronome (Robertson et al., 1999; Zelaznik et al., 2002). These spatial motor tasks have attracted particular attention in the motor timing field because they do not engage explicit timing mechanisms to achieve temporal goals (Robertson et al., 1999; Studenka and Zelaznik, 2008; Zelaznik et al., 2000, 2002). Instead, during drawing tasks, timing is thought to emerge implicitly from other motor control processes, such as those constraining the spatial trajectory (Turvey, 1977), with movement regularities assumed to be governed by the physical properties of self-organizing oscillatory systems (Kelso, 1995). This other mode of timing is referred to as “emergent” timing, in contrast to the working memory-based timing mechanisms described above, which correspond to an “explicit” mode of timing.

Recent evidence further suggests that the mode of timing recruited during a motor task is not solely determined by task type, i.e., finger tapping or circle drawing, but rather by an interaction between task characteristics and movement speed. Discrete or slow motor tasks primarily recruit explicit timing processes, whereas continuous or fast tasks favor emergent timing, with a cutoff around 2 Hz (or 0.5 s, Huys et al., 2008). Thus, a finger tapping task can exhibit emergent timing properties when performed at fast rates (Repp, 2008), while a circle drawing task can exhibit explicit timing properties when slowed down (Huys et al., 2010) or when intermittent pauses are introduced between cycles (Zelaznik et al., 2002). Altogether, these findings suggest that the mode of control can shift within the same motor task, particularly by manipulating the time interval between successive movements to make movement sequences more discrete or more continuous. Such temporal manipulation could also be used voluntarily as a strategy for motor inhibition, favoring one mode of motor control over the other depending on task constraints.

As a combination of finger tapping and circle drawing, the spatial tapping task requires participants to produce finger taps on visual targets arranged along a circular path (6 targets, ~60 taps per trial, or ten cycles) presented on a touchscreen. The tempo is set by a metronome and progressively decreases across trials (with interstimulus intervals ranging from 1.1 to 0.3 s). Participants are instructed to synchronize each tap with the auditory stimulus (primary goal) while also aiming to touch each spatial location (secondary goal). Comprehensive analyses of the timing and spatial properties of the task have been reported for each tempo in a previous study conducted on 70 young adults (Dione and Delevoye-Turrell, 2015). Timing analyses included measuring synchrony (i.e., whether the tap occurred at the same time as the beep), interval timing errors (i.e., whether the correct durations were produced), and autocorrelations of the time intervals and asynchronies, which indicate the mode of timing used, i.e., explicit or emergent. Spatial analyses consisted of computing spatial ellipses around the tap endpoints at each target location to reflect changes in the spatial trajectory. From these spatial ellipses, we measured the area as an indicator of spatial error, and the angles of the ellipses in reference to two trajectory models: alignment with the next target or with the global circle.

In summary, our results revealed a shift in timing mode from explicit to emergent from an interval of 0.7 s (autocorrelation analyses), with trajectories also starting to align with the global circle (angular deviation of ellipses), thus suggesting more continuous production at fast tempi. From 0.6 s, a specific spatial pattern emerged, with both spatial and timing errors reduced near the point of transition between the arm flexion and extension movement patterns (here, the north-west target), suggesting that anticipatory control occurred to smooth the trajectory. Spatial and interval timing errors were lowest at slow tempi, with a first increase in error observed from 0.8 s of interval. Errors then rose first slightly and then sharply (from 0.5 s for spatial error and 0.4 s for interval timing). Timing was predictive at slow tempi, with synchrony analyses revealing that the tap occurred on average before the beep until an interval of 0.6 s (included), and was slightly delayed at faster tempi (+0.02 s on average). Timing (interval errors and asynchronies) and spatial errors were correlated from 0.5 s, supporting the idea that timing was influenced by spatial constraints at these tempi, as expected when timing is emergent. Interval timing was further correlated with spatial error at an interval of 0.8 s, thus confirming the first change in constraints at this tempo. Altogether, this points to three main phases of motor control: (1) a slow phase (1.1 to 0.9 s), in which actions are produced discretely, i.e., with long pauses between taps, and timing is explicitly controlled and predictive; (2) an intermediate phase (0.8 to 0.6 s), in which an urge to produce faster, more continuous actions emerges and is probably inhibited to comply with the task instructions; and (3) a fast phase (0.5 to 0.3 s), in which actions are produced continuously, with an emerging need to control the movement trajectory, from which timing emerges.

The first aim of the present study is to explore whether the three EFs are engaged during the spatial tapping task as hypothesized: working memory during the slow/discrete phase, inhibition during the intermediate phase, and switching during the fast/continuous phase. To address this question, we will compare EF scores derived from the spatial tapping task with those obtained from a classical neuropsychological battery comprising three standardized tasks (TAP battery; Zimmermann and Fimm, 1994) in healthy young adults (*N* = 57). Our dataset combines data from two independent studies: an original study initially reported in conference proceedings (Dione et al., 2013) and a recent replication. Results will first be presented separately for each study to observe whether the findings were replicated; however, only the results from the combined dataset will be considered for interpretations. We predict that (1) lower interval timing error in the slow/discrete phase will be associated with greater working memory capacity, (2) longer contact time in the intermediate phase will be associated with greater inhibitory capacity, and (3) lower spatial error in the fast/continuous phase will be associated with greater switching capacity.

The second aim is to examine whether spatial tapping performance patterns obtained in clinical populations with executive deficits (aging, dementia, schizophrenia) are consistent with the EF impairments described in the literature, while also identifying potential confounding factors that may influence task performance, such as differences in motor output. Indeed, EF impairments often co-occur with low-level motor deficits, including psychomotor slowing in aging (Salthouse, 1996, 2000; Yordanova et al., 2004), and parkinsonism in schizophrenia (Caligiuri et al., 2019, 2022; Howes et al., 2023; Koning et al., 2010; Peralta et al., 2010), making their respective contributions difficult to disentangle. To examine the influence of motor changes on performance patterns, we will also present performance patterns from a group of handwriting experts, namely biscriptual individuals, who have learned to write in two distinct scripts (Arabic and Latin) since childhood. These individuals exhibit a motor advantage, writing faster and more legibly than monoscriptuals (Alhaddad et al., 2023, 2024, 2026), together with superior manual dexterity (Alhaddad et al., 2024).

We predict that: (1) in populations with executive deficits, the spatial tapping task should yield performance patterns that are either consistent with those reported in the EF literature or reveal more selective patterns of executive dysfunction. Indeed, the spatial tapping task differs from classical neuropsychological tasks in several ways: it is independent of semantic content (e.g., letters or numbers) and relies on the same visuo-auditory stimuli and task instructions regardless of the EF considered. This design may improve interpretability by reducing variability related to cultural, linguistic, or educational factors, while enhancing comparability through a more balanced assessment. Previous studies have shown that normal aging (Bherer et al., 2004; Charlot and Feyereisen, 2005; Clarys et al., 2009; Dempster, 1992; Grady, 2012; West, 1996), Alzheimer’s dementia (Weintraub and Wicklund, 2012) and schizophrenia (Bleich-Cohen et al., 2013; Carter et al., 1998; Glahn et al., 2005; Hemsley, 1976; Koike et al., 2013; Leung et al., 2013; Stadlbauer et al., 2013; Stevens et al., 2013; Yogev et al., 2003, 2004) affect each core EF. However, several studies suggest that working memory may be relatively preserved in schizophrenia (Frith and Done, 1989; Koike et al., 2013; Smith et al., 1998). We also predict that (2) the manual expertise developed by biscriptuals will help determine whether long-term differences in motor output per se contribute to spatial tapping performance beyond executive processes. This comparison should help clarify the extent to which motor factors contribute to task performance and, in turn, refine the interpretation of findings from clinical populations.

Altogether, we aim to explore the potential of the spatial tapping task to serve as a novel paradigm for investigating the three core EFs within the context of motor control and execution. Given the motor nature of the task, the contribution of low-level motor processes naturally raises questions. However, it is important to consider how such motor contributions may extend beyond the spatial tapping task and apply to any assessment involving a substantial motor response produced by the dominant hand. Future work should therefore focus on disentangling high-level executive processes from motor contributions, as well as from other potential factors (e.g., age-related loss of tactile sensitivity, Dione et al., 2023), in the spatial tapping task. Such efforts will be essential to further strengthen its potential for investigating EFs, thereby enhancing its applicability in both research and clinical settings.

## 2 Methods

### 2.1 Participants

Participants were healthy young adults (aged 21 ± 3, *N* = 115, 45 males), middle-aged adults (aged 50 ± 9, *N* = 20, 12 males), or older adults (aged 81 ± 9, *N* = 20, 6 males), individuals with Alzheimer’s dementia (aged 83 ± 4, *N* = 10, 1 male), schizophrenia (aged 44 ± 10, *N* = 20, 15 males) or biscriptuals (aged 23 ± 4, *N* = 31, 15 males). All participants were right-handed (self-reported or evaluated using the Edinburgh Handedness Inventory for the study on biscriptuality) and had normal or corrected-to-normal vision. Young, middle-aged adults and biscriptuals were recruited at University (Lille or Aix-Marseille, France), or through word of mouth. Older adults and individuals with Alzheimer’s dementia were recruited from medical retirement homes (Saint Remy, Notre-Dame du Bon Repos, Simon Benichou, Nancy, France) or via word of mouth. Inclusion criteria were based on medical history and a neuropsychological evaluation assessing the presence of a dysexecutive syndrome associated with memory impairment. All older adults included performed above the pathological threshold on each test and were overall healthy. Individuals with schizophrenia were recruited from a psychiatric day hospital or a sheltered workshop in Strasbourg, France. They were all medicated with antipsychotics and/or antidepressants, and those taking benzodiazepines were excluded due to their direct muscle-relaxant effects. All studies were conducted in accordance with the principles of the Declaration of Helsinki. Participants received an information sheet and provided written consent. When participants were under legal guardianship, the guardian was informed, and consent was co-signed. Data were initially collected in the context of specific studies, most of them were conducted in Lille between 2010 and 2013, except the study on biscriptuality, which was conducted in Marseille in 2022. Studies conducted before 2014 were approved by the local ethics committee for human sciences at the University of Lille 3. The most recent study was approved by the national French research ethics committee (RIPH2G: 22.00610.000116). Participants recruited before 2014 were not financially compensated. In the study on biscriptuality, participants received financial compensation.

### 2.2 Material, procedure and measurements: spatial tapping

For the spatial tapping task, participants were invited to stand in front of a touchscreen monitor (Elo Touch 19″ 1915 L) placed on a table and oriented parallel to its surface. However, to increase comfort, middle-aged adults, and older participants were invited to sit, with the screen set on a low table and tilted at an angle of 45°. A visual display composed of six black targets (10 mm in diameter; spaced 100 mm apart) arranged around a virtual circle with a radius of 100 mm was presented on the screen (for a photo of the set-up, see Dione and Delevoye-Turrell, 2015). Participants were instructed to tap each visual target in a counterclockwise sequence, starting from the southeastern one, using their right index finger with the fist closed, until the display disappeared (i.e., for at least 60 taps, corresponding to one trial). Note, however, that in the schizophrenia study, 8 schizophrenia individuals and 3 controls did the task in the clockwise direction due to an experimental error, which did not affect the results of the current study (i.e., no effect of movement direction was found for the variables measured in the present study). Participants were clearly informed that the goal was to synchronize their tapping as accurately as possible with an auditory metronome delivering regular tones (beep duration: 0.1 s; frequency: 440 Hz). The tempo varied across trials, decreasing stepwise from slow to fast: the inter-stimulus interval started at 1.1 s and decreased by 0.1 s increments down to 0.3 s, resulting in a total of nine trials. Participants who were standing were encouraged to keep their left arm relaxed along their body, while those sitting were instructed to rest their left arm on the left thigh. The task lasted less than 10 min. Visual display, auditory tones, and data collection were controlled using MATLAB.

Data were analyzed offline using custom Matlab programs. The series of taps were systematically checked for spatial outliers, such as touching unrelated locations on the screen, repeated taps on the same target, or spatial and temporal omissions. Errors and omissions were either removed or replaced with NaN values. Once pre-processed, dependent variables were computed. Interval timing error in the slow/discrete phase (1.1 to 0.9 s), contact time in the intermediate phase (0.8 to 0.6 s), and spatial error in the fast/continuous phase (0.5 to 0.3 s) were considered the three candidate measures of executive control during spatial tapping, hypothesized to recruit working memory, inhibition, and switching, respectively. Interval timing error was calculated as the absolute difference between the produced inter-response intervals and the expected inter-stimulus interval. The mean of these differences was then divided by the inter-stimulus interval and multiplied by 100 to obtain a percentage error. Spatial error was quantified as the mean area of spatial ellipses computed around the endpoints of each target, expressed in mm^2^. Contact time corresponded to the mean duration of touchscreen contacts, measured in seconds (s). After the average of these variables was computed for each trial, we verified whether participants were following the intended tempo. Trials deviating by more than ±0.05 s from the inter-stimulus interval were systematically discarded, as departures from the intended tempo could have biased the control towards another executive strategy. Performance scores were subsequently computed as the average across the three trials of each phase.

### 2.3 Material, procedure and measurements: EF battery

Three neuropsychological tests targeting each core EF were selected from the Test of Attentional Performance (TAP, Zimmermann and Fimm, 1994; Zimmermann and Fimm, 2004), which provides computerized assessments. Participants were seated in front of a computer. Task instructions were first provided verbally by the experimenter and then presented on the screen. A short familiarization trial was also conducted before each test. Depending on the task, participants were provided with one or two response keys. They were instructed to respond either with their right hand or with both hands, each placed on a respective key. For all tasks, participants were asked to respond as quickly as possible while maintaining a low error rate. The N-back task was used to assess working memory abilities, the Go/No-Go task to assess inhibition, and the Flexibility task to assess switching abilities.

In the N-back task, a series of single-digit numbers is presented sequentially in the center of the screen (100 items, including 15 targets). Participants are instructed to press the response key as quickly as possible whenever the current item is identical to the one presented two trials earlier. Performance was scored using the median reaction time for correct responses. In the Go/No-Go task, either a straight or a diagonal cross (“+” or “×”) briefly appears at the center of the screen, for a total of 40 trials. Participants are required to press the response key as quickly as possible when the diagonal cross (“×”) appears. The target stimulus is presented on 50% of the trials. Performance was scored according to the number of false alarms (i.e., responses to the straight cross). For the Flexibility task, two versions were used: one involving letters and numbers (Original Study), and another involving geometric shapes, either angular or rounded (Replication Study). Two stimuli were displayed simultaneously on the screen, and participants used two response keys. The task comprised two control conditions (or “pure blocks”) and one alternation condition. In the first control condition, participants pressed the response key corresponding to the same hemifield as the letter (e.g., left key for a letter presented on the left, and vice versa). In the second control condition, they responded to the number’s position in the same manner. In the alternation condition, participants alternated responses between letters and numbers from trial to trial, pressing the key located in the corresponding hemifield. Each control condition included 50 trials, and the alternation condition included 100 trials. Performance in the Flexibility task was scored using a switching cost, defined as the difference in reaction time between the averaged control conditions and the alternation condition (Rogers and Monsell, 1995). The neuropsychological testing session lasted approximately 1 hour.

### 2.4 Group designs

For the first part of the analyses, hereafter referred to as the “EF correlation analysis”, we combined data from an original study conducted between 2010 and 2011 with 25 healthy young adults (7 males; aged 19 ± 1 years, referred to as the Original Study) and a recent study conducted in 2022 with 32 healthy young adults (15 males; aged 23 ± 4 years, referred to as the Replication Study), resulting in a Combined Sample of 57 participants (22 males, aged 22 ± 4 years).

For the second part of the analyses, hereafter referred to as the “population analyses”, we included the original “dementia”, “schizophrenia”, and “biscriptual” groups along with their initially matched controls (paired by age and gender), and created additional groups from the controls to evaluate the effect of aging. To assess the effect of aging, we initially intended to compare young (≤30 years), middle-aged (31 to 64 years), and older adults (≥65 years). However, body posture (standing versus sitting) differed between the young adults and the other participants, and pre-analyses confirmed a significant impact on performance. Specifically, we compared performance scores between the 13 oldest participants from the young adult group and the 13 youngest participants from the middle-aged group and found a significant effect on spatial error, with error being higher when standing than when sitting (U = 30, *p* < 0.01). Because the age difference remained substantial (28 vs. 40 years), we also controlled for a potential age effect on spatial error. For this, we used data from all the young healthy adults who were standing (*N* = 113, mean age = 21, range 18–36 years) and found no correlation between age and spatial error (*R* = −0.042, ns.). Altogether, these results indicate that body posture per se had a significant effect on spatial error. Accordingly, no statistical comparisons between participants in standing versus sitting positions are conducted in subsequent analyses. To evaluate the effect of normal aging, we therefore compared middle-aged adults (aged 50 ± 8, *N* = 19, 12 males) to older adults (aged 81 ± 9, *N* = 20, 6 males), all of whom were seated. These two groups will hereafter be referred to as “adults” and “older”.

### 2.5 Statistical procedures

Data were analyzed using JASP (0.95.4.0). For the “EF correlation analysis”, we examined associations between performance scores from the spatial tapping task (interval timing error, contact time, spatial error) and those from the EF tasks (reaction times in the N-back task, false alarms in the Go/No-Go task, and switching cost in the Flexibility task). Correlations were first computed separately for the Original Study (*N* = 25) and the Replication Study 2 (*N* = 32), to examine whether the results were replicated, and then for the Combined Sample (*N* = 57), which constituted the main analysis. One-tailed correlations were used because we had specific directional hypotheses: (1) lower interval timing error in the slow/discrete phase should be associated with shorter reaction times in the N-Back task, indexing working memory capacity; (2) longer contact times in the intermediate phase should be associated with fewer false alarms in the Go/no-Go task, indexing inhibitory capacity; and (3) lower spatial error in the fast/continuous phase should be associated with lower switching costs in the Flexibility task, indexing switching capacity. The statistical summary reports, for each comparison, the number of observations included, the correlation coefficient (Pearson’s *r*), the *p-*value, the Vovk-Sellke maximum *p*-ratio (indicating the ratio of the likelihood of the *p*-value under H1 versus H0, or, in other words, how many times the *p*-value is more likely to occur under H1), the 95% confidence interval, the effect size (Fisher’s *z*), and its standard error.

For the “population analyses”, we conducted standard paired comparisons of performance scores obtained in the different populations, namely aging, dementia, schizophrenia, and biscriptuality, with their matched controls. For each comparison, normality was first assessed using the Shapiro–Wilk test. If the normality assumption was met, homogeneity of variances was evaluated with the Brown-Forsythe test. When both assumptions were satisfied, group means were compared using Student’s *t* test. If variances were unequal, Welch’s t test was applied (W). When the normality assumption was violated, the non-parametric Wilcoxon-Mann–Whitney test (U) was used. Effect sizes are reported as the Cohen’s *d* for parametric tests and as rank-biserial correlations for non-parametric tests. The alpha level was set to 0.05 for all analyses, as the hypotheses tested are specific to each population and do not involve comparison between populations.

## 3 Results

### 3.1 EF correlation analysis

The correlations between performance scores from the spatial tapping task and those from the EF battery are shown in Figure 1. Statistical details are provided in Table 1. In the Original Study (*N* = 25), all correlations were significant: lower interval timing error in the slow/discrete phase was associated with shorter reaction times in the N-back task, longer contact time in the intermediate phase was associated with fewer false alarms in the Go/No-Go task, and lower spatial error in the fast/continuous phase was associated with reduced switching costs in the Flexibility task. In the Replication Study (*N* = 32), these correlations were reproduced for all measures except inhibition, which nonetheless showed the expected negative association. In the Combined Sample (*N* = 57), all correlations were significant, even after removing the two more extreme values from the switching correlation (see Combined* in Table 1).

**Figure 1:**
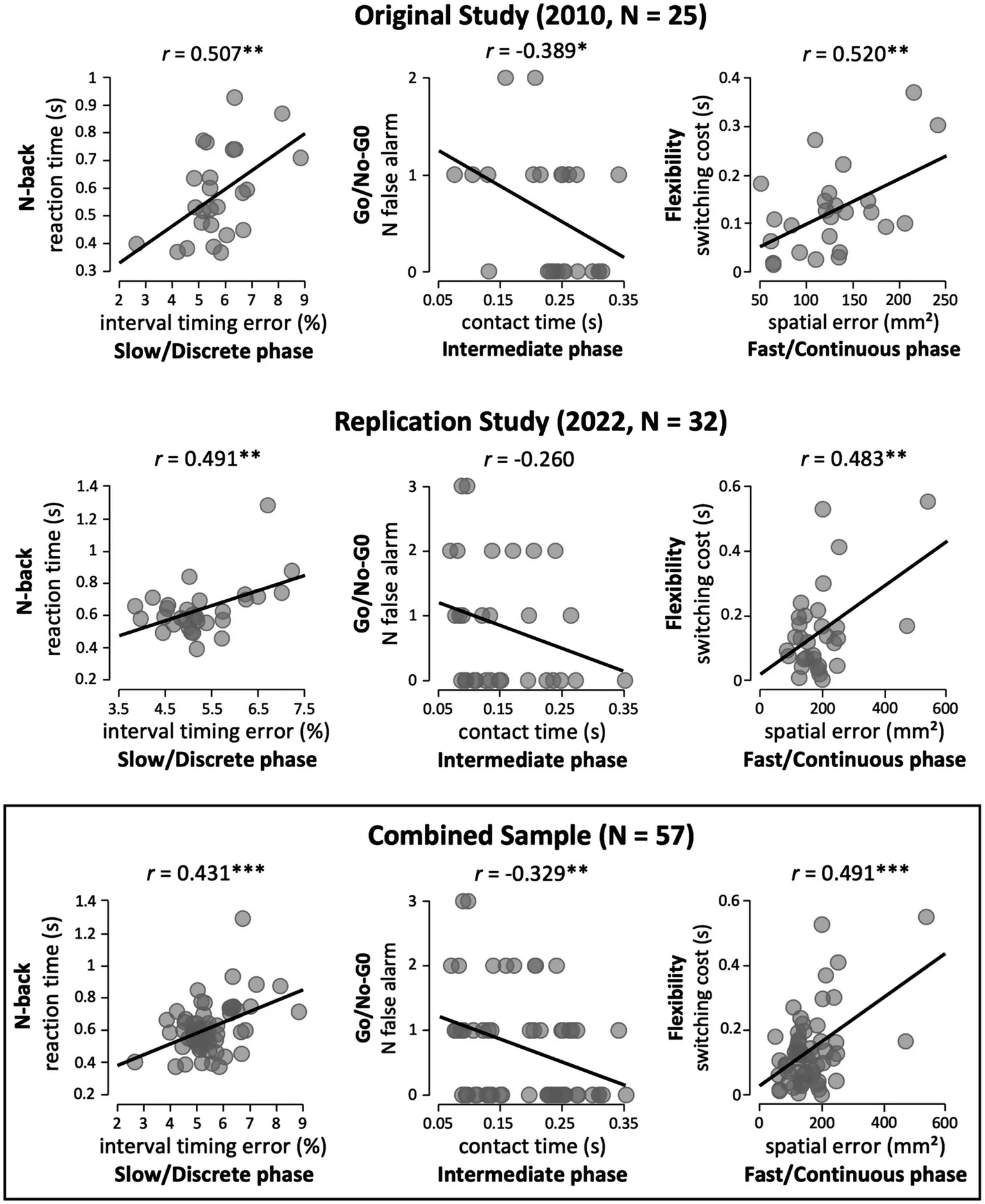
Scatter plots of the correlation results obtained in the Original Study (top panel), in the Replication Study (middle panel), and the Combined Sample (bottom panel), with the year of testing and number of participants specified in the panel titles. In each panel, from left to right, we can observe the correlations obtained for the working memory function, comparing interval timing error in the Slow/Discrete phase of the Spatial Tapping task with the reaction times in the N-Back task; the inhibition function, comparing contact time in the Intermediate phase of the Spatial Tapping task with the number of false alarms in the Go/No-Go task; and the switching function, comparing the spatial error in the Fast/Continuous phase of the Spatial Tapping task with the switching cost in the Flexibility task. Correlation values are indicated at the top of each plot, with asterisks indicating significance levels (*p < 0.05; **p < 0.01; ***p < 0.001). The alpha level is set to 0.05 for each test. Note that the Original and Replication studies are presented to provide descriptive details to the readers. The most relevant analysis is the Combined Sample, which is presented in a box to emphasize its importance.

**Table 1:** Summary of the correlation results, showing, for each comparison, the number of participants included (*n*), Pearson’s correlation coefficient (*r)*, the corresponding *p-*value (with significant results shown in bold; *α* = 0.05), the Vovk-Sellke maximum *p*-ratio, the 95% confidence interval, the effect size (Fisher’s *z*), and its standard error (SE).

Executive function	Spatial tapping	TAP	Study	*n*	*r*	*p*	VS-MPR	CI 95%	Effect size (SE)
Working memory	Slow/Disc.	N-back	Original	25	0.507	**0.005**	14.15	[0.204;1]	0.558 (0.213)
	*Interv. timing error*	*Reaction time*	Replication	30	0.491	**0.003**	21.40	[0.217;1]	0.537 (0.192)
			Combined	55	0.431	**<0.001**	94.72	[0.229;1]	0.461 (0.139)
Inhibition	Intermediate	Go/No-Go	Original	25	−0.389	**0.030**	3.491	[−1; −0.052]	−0.411 (0.218)
	*Contact time*	*False alarms*	Replication	30	−0.260	0.083	1.782	[−1; 0.051]	−0.266 (0.192)
			Combined	54	−0.329	**0.007**	10.03	[−1; −0.111]	−0.342 (0.140)
Switching	Fast/Cont.	Flexibility	Original	25	0.520	**0.004**	17.05	[0.221;1]	0.576 (0.213)
	*Spatial error*	*Switching cost*	Replication	31	0.483	**0.003**	21.20	[0.212;1]	0.526 (0.189)
			Combined	56	0.491	**<0.001**	615.4	[0.301;1]	0.537 (0.137)
			Combined*	54	0.338	**0.006**	11.74	[0.121;1]	0.352 (0.140)

*No outlier.

### 3.2 Population analyses

The results of the population analyses are presented in Figure 2.

**Figure 2:**
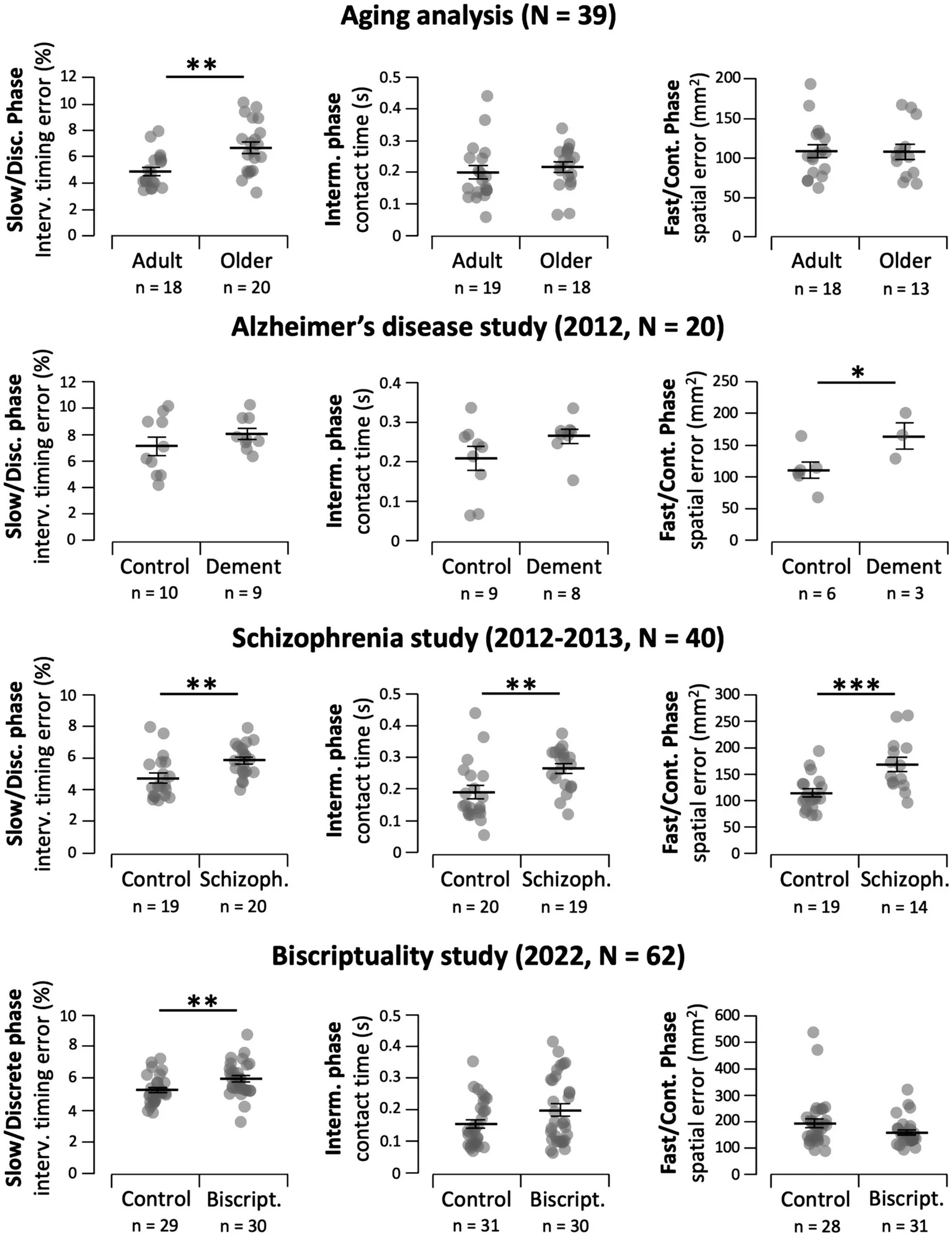
Performance scores obtained in the Spatial Tapping task are presented for each separate analysis, with, from top to bottom, the aging analysis, the Alzheimer’s disease study, the schizophrenia study, and the biscriptuality study. The year of testing and number of participants initially included are specified in the panel titles where relevant. Aging was not a study per se; data were recombined from the entire dataset to enable this analysis. Performance scores for each clinical or expert group are compared with those of matched controls (matched for gender and age where relevant). Each panel presents three group comparison plots, showing, from left to right, interval timing error in the Slow/Discrete phase, contact time in the Intermediate phase, and spatial error in the Fast/Continuous phase. In each plot, individual scores are shown in the background, while means and standard errors are displayed in the foreground. Horizontal comparison lines with asterisks above indicate significant differences between groups (*p < 0.05; **p < 0.01; ***p < 0.001).

*Effect of aging.* There was a significant effect of aging on interval timing error, with older adults showing higher percentage error (mean = 6.7%) than middle-aged adults (mean = 4.9%, *t*(36) = −3.249, *p* = 0.003, effect size = −1.056, SE effect size = 0.369). No significant effect was observed for contact time (mean = 0.205 s, *t*(35) = −0.616, *p* = 0.542, effect size = −0.203, SE effect size = 0.331) or spatial error (mean = 109 mm^2^, U = 121, *p* = 0.890, effect size = −0.034, SE effect size = 0.210).

*Effect of Alzheimer’s dementia.* Dementia had no significant effect on interval timing error (mean = 7.6%, *t*(17) = −1.121, *p* = 0.278, effect size = −0.515, SE effect size = 0.474) or contact time (mean = 0.236 s, *t*(15) = −1.530, *p* = 0.147, effect size = −0.743, SE effect size = 0.517). However, spatial error was significantly affected (*t*(7) = −2.354, *p* = 0.051, effect size = −1.664, SE effect size = 0.855).

*Effect of schizophrenia.* Schizophrenia significantly affected interval timing error, with schizophrenia individuals showing higher error (mean = 5.8%) than their paired controls (mean = 4.7%, U = 89, *p* = 0.005, effect size = 0.532, SE effect size = 0.185). Contact time was also longer in schizophrenia individuals (mean = 0.262 s) compared to controls (mean = 0.189 s, *t*(37) = −2.81, *p* = 0.008, effect size = −0.899, SE effect size = 0.351), and spatial error was higher (mean = 167 mm^2^ vs. 113 mm^2^, t(31) = −3.709, *p* < 0.001, effect size = −1.306, SE effect size = 0.411).

*Effect of biscriptuality.* Biscriptuals exhibited higher interval timing error (mean = 6%) than their paired controls (mean = 5.3%, *t*(57) = −2.751, *p* = 0.008, effect size = −0.716, SE effect size = 0.277), while no significant effects were found for contact time (mean = 0.177 s, U = 575.5, *p* = 0.113, effect size = −0.238, SE effect size = 0.148) or spatial error (mean = 177 mm^2^, U = 324, *p* = 0.097, effect size = 0.253, SE effect size = 0.151).

## 4 Discussion

In the present study, we explored whether the three core EFs, working memory, inhibition, and switching, are involved in the spatial tapping task, using comparisons of scores with classic neuropsychological tests and evidence from clinical or expert populations. First, we examined the associations between each EF and task performance, which supported our predictions: working memory performance was associated with the control of interval timing in the slow/discrete phase, inhibition with the induction of longer pauses between gestures at intermediate tempi, and switching with the control of spatial trajectories in the fast/continuous phase. Second, we investigated how performance scores in the spatial tapping task varied in populations with executive and motor impairments (aging, Alzheimer’s dementia, and schizophrenia) compared with their matched controls, to examine whether these patterns were consistent with the executive deficits described in the EF literature. We also included a population with a reported motor advantage in writing (biscriptuals), but without expected executive deficits, to evaluate and discuss the influence of changes in motor output on performance scores. Individuals with executive deficits were consistently impaired in the spatial tapping task, in ways that were either comparable (in schizophrenia) or more specific (in aging, dementia) than those reported in the EF literature. Furthermore, the motor advantage observed in biscriptuals also affected a performance measure (interval timing error), supporting the contribution of motor output to performance. These findings highlight both the potential of the task as a paradigm for investigating core EFs and the importance of disentangling executive and non-executive components to improve its specificity and applicability in both research and clinical settings. The potential contribution of sensory impairments (e.g., touch and proprioception) is discussed as well, paving the way for future development of the paradigm into a more global platform for sensorimotor evaluation, potentially enabling executive, motor, and sensory effects to be dissociated into separate sub-scores.

The spatial tapping task is designed to engage each core EF within a single paradigm, with executive control varying as a function of tempo phases that favor either discrete productions with long pauses between movements or continuous motion. In the slow/discrete phase (1.1 s to 0.9 s), timing control is assumed to be explicit, relying on clock mechanisms (Creelman, 1962; Gibbon et al., 1984; Treisman, 1963), thereby engaging working memory (Bååth et al., 2016; Broadway and Engle, 2011a, 2011b; Holm et al., 2017; Maes et al., 2015; Ogden et al., 2011). In the fast/continuous phase (0.5 s to 0.3 s), timing control is assumed to emerge from the movement dynamics constraining the spatial trajectory (Huys et al., 2008; Robertson et al., 1999; Turvey, 1977; Zelaznik et al., 2002), rather than being explicitly processed. Switching control is assumed to be required to facilitate circular motion by controlling the transitions between flexion and extension patterns of arm movement, thereby reducing the spatial error near transition points (Dione and Delevoye-Turrell, 2015; Roerdink et al., 2008). At intermediate tempi (0.8 to 0.6 s), inhibitory mechanisms are assumed to be required to keep actions discrete, i.e., with long pauses between successive movements, thereby favoring explicit timing despite an emerging urge to produce faster and more continuous actions and preventing a shift toward emergent timing control. This is assumed to occur through longer pauses between successive movements, as reported for other hybrid timing tasks combining explicit and emergent constraints (Huys et al., 2008; Repp, 2008; Zelaznik et al., 2002).

In the present study, we aimed to explore whether the three core EFs are engaged as hypothesized in the spatial tapping task, i.e., with working memory in the slow/discrete phase, inhibition in the intermediate phase, and switching in the fast/continuous phase. To this end, we invited healthy young adults to perform both the spatial tapping task and a battery of three executive tasks evaluating the core EFs. These relationships between EF performance and spatial tapping performance were examined in a previous study (see Dione et al., 2013, for conference proceedings), here referred to as the Original Study, and were recently reassessed in a different context (handwriting) using the exact same setup but with different experimenters, here referred to as the Replication Study. Notably, the selection of the TAP (Zimmermann and Fimm, 1994; Zimmermann and Fimm, 2004) was made independently by the first author of the handwriting study (AG), who is also a co-author of the current paper. This provided an opportunity to independently reassess assumptions underlying spatial tapping, which are reported here for the first time.

In the Original Study, we found the expected associations for each core EF. In the Replication Study, the results were replicated for working memory and switching, but not for inhibition. Nevertheless, the negative association was present, although it did not reach statistical significance (*p* = 0.083). Altogether, the relationship with the Go/No-Go task was the weakest in both studies. This may reflect the lack of sensitivity of the Go/No-Go task, in which false alarms (i.e., pressing the key for the wrong cross) ranged from 0 to 3 in our groups of healthy young adults. It is also possible that, during spatial tapping, inhibition is not limited to contact with the touchscreen but also occurs during flight times (Bieńkiewicz et al., 2012), thereby reducing the strength of the measure. This could be addressed in a future study either by using inhibition tasks other than the Go/No-Go task or by analyzing entire movement trajectories alongside touchscreen data during spatial tapping (e.g., using motion capture).

One can also question the association found between the switching cost of the Flexibility task and the spatial error in the fast/continuous phase of the spatial tapping task. Indeed, two data points appear to strongly influence the correlation, as shown in Figure 1, and also emerged as potential outliers in the group results of the population analyses (see the controls in the Biscriptuality study in Figure 2). However, the association remained significant when these two points were removed from the analysis (*r* = 0.338, *p* = 0.006; see Table 1 for more details). We also verified whether spatial errors were overall higher in this group by comparing scores with those from a group of controls matched for age and gender, randomly selected from the entire group of healthy young controls (N = 115), while excluding the two potential outliers. Results confirmed a more general effect, with significant differences between the two groups (mean = 120 vs. 173 mm^2^, W(50.92) = −4.66, *p* < 0.001). We then examined whether these differences could be explained by the different stimuli used in the Flexibility task, i.e., letters and numbers in the Original Study versus round and angular shapes in the Replication Study by examining the norms in the TAP. However, we found the reverse effect in these cohorts (aged 20 to 90, including 158 to 810 participants depending on the condition), i.e., shorter switching costs with geometric shapes than with letters/numbers (0.2 s versus 0.4 s, when recalculated from the mean reaction times proposed for each subtest, i.e., control or alternation conditions). The difference in stimuli is therefore unlikely to be the main cause of the observed differences. Another possibility is that the large number of writing tasks that participants completed in this study (Alhaddad et al., 2024) may have promoted more continuous motor production during the spatial tapping task. Similar effects have been reported in tasks requiring or not requiring ‘self-control’, the former influencing the latter (see the discussion in Diamond, 2013; Muraven, 2010; Muraven and Baumeister, 2000). Another explanation could be that the temporal gap of nearly a decade between the two studies introduced a generational effect, with a decline in fine motor skills (Sülzenbrück et al., 2011) and EF performance (Cui et al., 2024; Liu et al., 2022; Maeneja et al., 2025) in younger generations, possibly due to increasing use of computers for producing written texts (Sülzenbrück et al., 2011), increased screen time (Liu et al., 2022; Maeneja et al., 2025) and more sedentary behaviors (Cui et al., 2024).

From another perspective, one could also argue that the strength of the associations was overall slightly reduced in the Replication Study, with the weakest association falling below the significance threshold. Such decreases in association are commonly reported in replication studies, and borderline replications are often still considered valid (Aarts et al., 2015). In the cited study, in which 100 psychology studies were retested to evaluate replication, only 39% were replicated, including borderline studies, while 68% remained significant after combining the original and replicated results. Accordingly, the results we obtained in the present study, i.e., weaker associations in the Replication Study, together with the finding that all associations were significant in the Combined Sample, provide support for our assumption. Considering the differences between studies was important in the context of the replication, but for interpreting the results, the analysis of interest is that of Combined Sample (*N* = 57). Accordingly, we can conclude that (1) lower interval timing error in the slow/discrete phase of spatial tapping is associated with shorter reaction times in the N-back task reflecting greater working memory capacity, (2) longer contact time in the intermediate phase is associated with lower number of false alarms in the Go/No-Go task, reflecting greater inhibitory capacity, (3) lower spatial error is associated with smaller switching costs in the Flexibility task, reflecting greater switching capacity. These results provide initial evidence that the three core EFs may be involved in control during spatial tapping.

Similar associations or more causal experimental approaches (e.g., dual-tasks paradigms) between motor timing tasks and core EFs have been conducted previously, with however a focus on explicit timing tasks (Bååth et al., 2016; Broadway and Engle, 2011a, 2011b; Fortin et al., 2010; Fortin and Schweickert, 2016; Holm et al., 2017; Maes et al., 2015; Ogden et al., 2011; Viau-Quesnel and Fortin, 2014; Zakay and Shub, 1998). These studies confirmed that working memory is recruited when timing is explicit (Bååth et al., 2016; Broadway and Engle, 2011a, 2011b; Holm et al., 2017; Maes et al., 2015; Ogden et al., 2011), but is not recruited when timing is emergent or fast (Bååth et al., 2016; Maes et al., 2015). The other two EFs, namely inhibition and switching, have not been found to contribute to explicit timing tasks (Fortin et al., 2010; Fortin and Schweickert, 2016; Ogden et al., 2011; Viau-Quesnel and Fortin, 2014; Zakay and Shub, 1998). Thus, to our knowledge, this is the first study to examine and report significant associations of inhibition and switching with performance in a motor timing task with spatial constraints. However, as our evidence is based on correlations, these findings require confirmation using more direct approaches in future studies.

Introducing a motor task to assess executive functioning is not a trivial choice. The importance of EFs in organizing actions and activities has been emphasized since the earliest neuropsychological investigations (Luria, 1966; Norman and Shallice, 1986; Shallice, 1982, 1988). Motor tasks such as sequential tapping tasks were used in the early investigations (Luria, 1966), and driving a car has been a classic example used to illustrate executive functioning (Shallice, 1988). The most commonly used EF tasks, such as the Tower of London (Shallice, 1982) and the Wisconsin Card Sorting Test (WCST; Grant and Berg, 1948), require participants to move colored beads across pegs to match a target configuration in as few moves as possible, or to sort cards according to an implicit rule that changes unpredictably, requiring participants to adjust their responses based on feedback. Thus, these tasks all involve motor responses that must be organized and controlled in accordance with task goals (e.g., minimizing the number of moves or sorting cards to maximizing the number of correctly identified sorting rules). Inhibition tasks most often require restraining a motor response, and switching tasks are often bimanual, thus requiring shifting control across different muscle groups (see Diamond, 2013; Miyake et al., 2000, for examples of tasks). Overall, the spatial tapping task incorporates key features of standard EF tasks: movement control and execution, timing goals and spatial constraints, while not requiring language and employing a balanced set of stimuli across conditions assumed to engage EFs.

It is also important to acknowledge that spatial tapping task is not simply a motor task: it requires cognitive processes to organize sequences of movements in space and time. The temporal and spatial organization of behavior are fundamental aspects of cognition, yet they are rarely evaluated per se. They also play a recognized role in executive functioning, although their contribution remains underappreciated. Indeed, timing is closely involved in both inhibition and working memory. Developmental studies have reported that children improve their capacity to inhibit responses and select the correct response when time delays are introduced between the stimulus and the expected response (Diamond, 2002; Simpson et al., 2012; Simpson and Riggs, 2007). A visual stimulus (e.g., a candy) strongly evokes a prepotent response (e.g., grasping and eating it), which becomes weaker when a significant delay is imposed, allowing the correct response to be selected instead (e.g., refraining from eating the candy). Working memory involves maintaining information in mind over a period of time and using it to guide decisions or monitor actions. Recent work has reported that timing and working memory can be encoded through shared neural signatures, suggesting an overlap in the neural mechanisms supporting these processes (Zhou et al., 2023). The switching function has been described as requiring the ability to change spatial perspectives, explore other directions, or think outside the box, in other words, to develop multiple mental trajectories and flexibly shift between them (Diamond, 2013). Although metaphorical, this definition highlights potential contribution of spatial processing to task switching. Furthermore, brain regions involved in spatial processing and navigation, such as the hippocampus (O’Keefe and Dostrovsky, 1971), also play a recognized role in task switching (Schmidt et al., 2020). Our view overall supports the idea that EFs, and cognition more broadly, are not constructs that develop independently of the rest of the body. Rather, they can be understood as emerging from the need to organize complex behavior in ways that enable individuals to interact adaptively with their surrounding environment and deal with novel situations (Koziol and Lutz, 2013).

The second aim of the present study was to examine the consistency of the performance patterns obtained in clinical populations with the EF impairments described in the literature. We hypothesized that design features, namely independence from semantic content, the balanced use of visuo-auditory stimuli throughout task, and the use of a single, simple instruction, would improve the interpretability and comparability of EF-related scores, yielding more specific patterns in populations with presumed global EF impairments (normal aging, dementia, and schizophrenia). In line with this hypothesis, if one assumes that the EFs are engaged as expected in the spatial tapping task, more specific impairments were found in aging and dementia, whereas results in schizophrenia were comparable, i.e., consistent with a global impairment. In aging, interval timing error was the only measure affected, consistent with a specific working memory deficit rather than a global EF impairment. In Alzheimer’s dementia, spatial error was selectively impaired, consistent with a specific switching deficit. In schizophrenia, all performance scores were affected, thus consistent with the global EF impairment described in the literature. Note, however, that contact times were longer than in controls, which may instead reflect over-engagement of inhibition during this phase, rather than a lack of inhibitory capacity, which would presumably result in shorter contact times.

We also analyzed the performance scores of a population of biscriptuals, demonstrating motor expertise in writing (Alhaddad et al., 2023, 2024, 2026), without any expected differences in executive functioning. During spatial tapping, we found that interval timing was increased compared to controls, which would be consistent with a working memory deficit. However, there is no reason to assume that working memory per se is impaired in biscriptuals. Indeed, using a N-back task and the Corsi block-tapping task in the same group of biscriptuals, we found no differences in working memory compared with monoscriptuals (Alhaddad et al., 2024). This result may instead reflect the consequences of their lifelong writing experience, which may have constituted a form of overtraining for producing fast, continuous movements, favoring a general tendency to act quickly. Such training is not consistent with the task requirements for interval timing, which require movements to be slowed down. Thus, because the task was novel for them, it required skills opposite to those they have developed (i.e., slowing down movements), and was performed only once, they may have had greater difficulty adapting their behavior to these constraints. In other words, this may represent a case of “negative transfer of learning”, as reported in athletes (Oppici and Panchuk, 2022; Schmidt and Lee, 2011), and repeated exposure to the task might therefore be expected to improve their performance. This result highlights the importance of considering the multiple components that may affect performance scores during spatial tapping, here illustrating the contribution of motor output per se, i.e., how muscles are activated independently of the task goal. Thus, the variables proposed here cannot be straightforwardly interpreted as reflecting the executive component: even if they are associated with EF scores, alternative explanations must be considered, and further research is needed to dissociate the different sources of variance contributing to these scores.

Let us consider the most relevant alternative explanations for the other groups evaluated in the current study in light of current knowledge of these populations. Aging, considered in the current study from adulthood (50 years) to advanced age (81 years), affected interval timing performance, with more errors being produced with age. Although this could reflect a working memory deficit in aging, the interval timing deficit could also result from psychomotor slowing associated with aging, which is a recognized confounding factor in cognitive testing, including EF tasks (Salthouse, 1996, 2000). Psychomotor slowing affects both central processing and motor execution (Salthouse, 1996, 2000; Yordanova et al., 2004) and has been associated with alterations in neural conduction often interpreted as reflecting the normal aging of the nervous system (Gunning-Dixon and Raz, 2000; Kennedy and Raz, 2009). Central slowing could have directly affected the internal clock by slowing its pace and resulting in altered time perception, thereby affecting performance. Peripheral slowing could result in slower, weaker or overall more variable muscle activations, thus directly affecting the timing of the produced movement. With aging, there is also a decline in tactile sensitivity (Dione et al., 2023; Infante et al., 2025; Samain-Aupic et al., 2024; Skedung et al., 2018) and proprioception (Chancel et al., 2018; Landelle et al., 2021), which could also affect timing performance, by reducing the perception of the moment of contact and, more generally, the perception of the movements being produced.

In schizophrenia, motor impairments are substantial (Koning et al., 2010; Peralta et al., 2010) and increase with antipsychotic medication (Caligiuri et al., 2019, 2022; Howes et al., 2023). They are described as a form of parkinsonism, including bradykinesia, characterized by motor slowing, rigidity, and difficulties initiating movements or executing actions with the correct amplitude (Caligiuri et al., 2019, 2022; Koning et al., 2010), as well as dyskinesia (Koning et al., 2010), tremor, or catatonia (Peralta et al., 2010). These motor impairments could have affected all three performance variables in spatial tapping without reflecting an EF impairment. This alternative interpretation would be consistent with findings from several studies showing that working memory is preserved to at least some degree in schizophrenia (Frith and Done, 1989; Koike et al., 2013; Smith et al., 1998). In the study by Frith and Done (1989), it was proposed more specifically that individuals with schizophrenia are impaired in using sensory feedback to monitor their actions (Frith and Done, 1989). Difficulties in integrating visual information and motor actions have also been proposed (Giersch et al., 2013). Longer contact times have also been reported in spontaneous tapping tasks, which are assumed not to require executive control (Delevoye-Turrell et al., 2012), and as such, could instead reflect difficulties in initiating movements or motor slowing. Altogether, the significant impact of motor impairments in schizophrenia makes the interpretation of EF-related scores difficult, if not impossible, in this population. In the future, it could be interesting to compare individuals with schizophrenia on and off medication to further isolate the impact of motor impairments on spatial tapping performance variables.

For individuals with Alzheimer’s dementia, it is important to consider that we are only reporting preliminary results. Indeed, we could only recruit 10 individuals, of whom only 3 completed the task correctly at fast speeds. Following the tempo is an important requirement in spatial tapping, as slower speeds may engage alternative executive control strategies, thus biasing the evaluation. Despite the low number of participants, we report a specific impairment in spatial error, which is consistent with an impairment in the switching function. This potential effect actually aligns with the progressive neurodegeneration affecting the brain in this pathology, primarily affecting hippocampal regions (Braak and Braak, 1991; Planche et al., 2022), which have also been implicated in task switching (Schmidt et al., 2020). Alternative explanations also exist, such as difficulties in integrating visual information to guide motor responses and impairments in eye-hand coordination in this pathology (Lu et al., 2021; Nardone et al., 2022; Tippett and Sergio, 2006; Verheij et al., 2012), associated with difficulties maintaining the correct amplitude when tracing lines (Tippett and Sergio, 2006) or drawing circles (Lu et al., 2021).

The data presented in the present study are a compilation of all data collected with the spatial tapping task since its inception. The objective is to revive the task, as well as to identify its limitations and address them in future work. The most important limitation was that the body posture differed between the young adults, who were standing, and the other participants, who were seated with the screen tilted, which prevented comparison with the younger participants, as we suspected an important effect of body posture. The effects of visual display size and positioning require further investigation, especially to enable flexible use of the task on modern touchscreen devices in the future. Participants performed only one trial per tempo and the analysis was conducted offline. Thus, the experimenter was not always aware that participants were not following the tempo, and could not decide to repeat the trial accordingly, which largely explains the loss of data. Similarly, some participants performed the task in the wrong direction (clockwise) due to an experimenter error. These latter limitations could be addressed in the future by improving the task programming, including the provision of performance feedback.

To conclude, in the present study, we provided preliminary evidence that the spatial tapping task has the potential to engage the three core EFs within the context of motor control. It is therefore an ecological task, rooted in action and execution, that enables a balanced assessment (same instruction, same visuo-auditory content throughout the task), is fast to perform (10 min), independent of language, and easy to explain with a single instruction throughout the task. Future research should include improving our understanding of the sub-components contributing to the final performance scores in order to dissociate their respective contributions, including components such as motor execution, touch, proprioception, vision, timekeeping mechanisms, and sensorimotor integration. Such work could advance our understanding of executive functions and sensorimotor control, as well as provide further insight into the subtleties of the associated pathologies.

## Data Availability

The datasets presented in this study can be found in online repositories. The names of the repository/repositories and accession number(s) can be found at: Zenodo platform, https://doi.org/10.5281/zenodo.22083340.

## References

[ref1] Aarts A. A. Anderson J. E. Anderson C. J. Attridge P. R. Attwood A. Axt J. . (2015). Estimating the reproducibility of psychological science. Science 349:aac4716. doi: 10.1126/SCIENCE.AAC4716 26315443

[ref2] Alhaddad G. Danna J. Gilhodes J.-C. Younes-Harb C. Longcamp M. (2026). The Imprint of Biscriptuality on Planning and Online Control of Handwriting (Preprint Accessible at SSRN). doi: 10.2139/SSRN.6116167

[ref3] Alhaddad G. Danna J. Younes-Harb C. Velay J.-L. Longcamp M. (2023). Effects of biscriptuality on graphomotor coordination dynamics. J. Exp. Psychol. Hum. Percept. Perform. 49, 177–187. doi: 10.1037/xhp0001075, 36455050

[ref4] Alhaddad G. Dione M. C. Danna J. Alario F. A. Honnorat Velay J.-L. . (2024). Writing in two different scripts promotes fine motor control. Cortex 179, 247–260. doi: 10.1016/j.cortex.2024.07.016 39213777

[ref5] Bååth R. Tjøstheim T. A. Lingonblad M. (2016). The role of executive control in rhythmic timing at different tempi. Psychon. Bull. Rev. 23, 1954–1960. doi: 10.3758/s13423-016-1070-1, 27301408

[ref6] Bherer L. Belleville S. Hudon C. (2004). Executive function deficits in normal aging, Alzheimer’s disease, and frontotemporal dementia. Psychol. Neuropsychiatr. Vieil. 2, 181–189. 15689332

[ref7] Bieńkiewicz M. Rodger M. Craig C. (2012). Timekeeping strategies operate independently from spatial and accuracy demands in beat-interception movements. Exp. Brain Res. 222, 241–253. doi: 10.1007/s00221-012-3211-8, 22903462

[ref8] Bleich-Cohen M. Hendler T. Weizman R. Faragian S. Weizman A. Poyurovsky M. (2013). Working memory dysfunction in schizophrenia patients with obsessive-compulsive symptoms: an fMRI study. Eur. Psychiatry 29, 160–166. doi: 10.1016/j.eurpsy.2013.02.004, 23764406

[ref9] Braak H. Braak E. (1991). Neuropathological stageing of Alzheimer-related changes. Acta Neuropathol. 82, 239–259. doi: 10.1007/BF00308809, 1759558

[ref10] Broadway J. M. Engle R. W. (2011a). Individual differences in working memory capacity and temporal discrimination. PLoS One 6:e25422. doi: 10.1371/journal.pone.0025422, 22003391 PMC3189201

[ref11] Broadway J. M. Engle R. W. (2011b). Lapsed attention to elapsed time? Individual differences in working memory capacity and temporal reproduction. Acta Psychol. 137, 115–126. doi: 10.1016/j.actpsy.2011.03.008, 21470583

[ref12] Brown S. W. (2006). Timing and executive function: bidirectional interference between concurrent temporal production and randomization tasks. Mem. Cogn. 34, 1464–1471. doi: 10.3758/BF03195911, 17263071

[ref13] Burgess P. W. Alderman N. Evans J. Emslie H. Wilson B. A. (1998). The ecological validity of tests of executive function. J Int Neuropsychol Soc 4, 547–558. doi: 10.1017/S1355617798466037, 10050359

[ref14] Caligiuri M. P. Teulings H. L. Dean C. E. Lohr J. B. (2019). The nature of bradykinesia in schizophrenia treated with antipsychotics. Psychiatry Res. 273, 537–543. doi: 10.1016/j.psychres.2019.01.058, 30710809 PMC6561794

[ref15] Caligiuri M. P. Weiden P. J. Legedza A. Yagoda S. Claxton A. (2022). Handwriting kinematics in patients with schizophrenia treated with long-acting injectable atypical antipsychotics: results from the ALPINE study. Schizophr. Bull. Open 3:sgac018. doi: 10.1093/schizbullopen/sgac018, 39144789 PMC11205961

[ref16] Carter C. S. Perlstein W. Ganguli R. Brar J. Mintun M. Cohen J. D. (1998). Functional hypofrontality and working memory dysfunction in schizophrenia. Am. J. Psychiatry 155, 1285–1287. doi: 10.1176/ajp.155.9.1285, 9734557

[ref17] Chancel M. Landelle C. Blanchard C. Felician O. Guerraz M. Kavounoudias A. (2018). Hand movement illusions show changes in sensory reliance and preservation of multisensory integration with age for kinaesthesia. Neuropsychologia 119, 45–58. doi: 10.1016/J.NEUROPSYCHOLOGIA.2018.07.027, 30063911

[ref18] Charlot V. Feyereisen P. (2005). Episodic memory, inhibition deficit, and cognitive aging: an examination of the frontal hypothesis. Annee Psychol. 2, 323–357. doi: 10.3406/psy.2005.29699

[ref19] Clarys D. Bugaiska A. Tapia G. Baudouin A. (2009). Ageing, remembering, and executive function. Memory (Hove, England) 17, 158–168. doi: 10.1080/09658210802188301, 18615347

[ref20] Creelman C. D. (1962). Human discrimination of auditory duration. J. Acoust. Soc. Am. 34, 582–593. doi: 10.1121/1.1918172

[ref21] Cui J. Mu J. L. Liao J. J. Shi Z. Y. Qin K. P. Li L. (2024). Association of screen-based sedentary behavior with executive function in school-aged children: a functional near-infrared spectroscopy study. J. Exp. Child Psychol. 244:105956. doi: 10.1016/j.jecp.2024.105956, 38735222

[ref22] Damasio A. R. (1994). Descartes’ Error: Emotion, Rationality and the Human Brain. New York, NY: Random House.

[ref23] Delevoye-Turrell Y. Wilquin H. Giersch A. (2012). A ticking clock for the production of sequential actions: Where does the problem lie in schizophrenia? Schizophr. Res. 135, 51–54. doi: 10.1016/j.schres.2011.12.020, 22260961

[ref24] Dempster F. N. (1992). The rise and fall of the inhibitory mechanism: toward a unified theory of cognitive development and aging. Dev. Rev. 12, 45–75. doi: 10.1016/0273-2297(92)90003-K

[ref25] Diamond A. (2002). “Normal development of prefrontal cortex from birth to young adulthood: cognitive functions, anatomy, and biochemistry,” in Principles of Frontal Lobe Function, eds. Stuss D. Knight R. (Oxford: Oxford Univ. Press).

[ref26] Diamond A. (2013). Executive functions. Annu. Rev. Psychol. 64, 135–168. doi: 10.1146/ANNUREV-PSYCH-113011-143750, 23020641 PMC4084861

[ref27] Dione M. C. Delevoye-Turrell Y. (2015). Testing the co-existence of two timing strategies for motor control in a unique task: the synchronisation spatial-tapping task. Hum. Mov. Sci. 43, 45–60. doi: 10.1016/j.humov.2015.06.009, 26203523

[ref28] Dione M. C. Ott L. Delevoye-Turrell Y. (2013). “Planning ahead through space and time: from neuropsychology to motor control,” in Proceedings of the 35 th Annual Conference of the Cognitive Science Society, eds. Knauff M. Pauen M. Sebanz N. Wachsmuth I.. 2207–2212.

[ref29] Dione M. C. Watkins R. Aimonetti J. Jourdain R. Ackerley R. (2023). Effects of skin moisturization on various aspects of touch showing differences with age and skin site. Sci. Rep. 13:17977. doi: 10.1038/s41598-023-44895-w, 37863946 PMC10589338

[ref30] Fortin C. Schweickert R. (2016). Timing, working memory and expectancy: a review of interference studies. Curr. Opin. Behav. Sci. 8, 67–72. doi: 10.1016/j.cobeha.2016.01.016

[ref31] Fortin C. Schweickert R. Gaudreault R. Viau-Quesnel C. (2010). Timing is affected by demands in memory search but not by task switching. J. Exp. Psychol. Hum. Percept. Perform. 36, 580–595. doi: 10.1037/a0017639, 20515190

[ref32] Friedman N. P. Miyake A. (2017). Unity and diversity of executive functions: individual differences as a window on cognitive structure. Cortex 86, 186–204. doi: 10.1016/j.cortex.2016.04.023, 27251123 PMC5104682

[ref33] Frith C. D. Done D. J. (1989). Experiences of alien control in schizophrenia reflect a disorder in the central monitoring of action. Psychol. Med. 19, 359–363. doi: 10.1017/S003329170001240X, 2762440

[ref34] Gibbon J. Church R. M. Meck W. H. (1984). Scalar timing in memory. Ann. N. Y. Acad. Sci. 423, 52–77. doi: 10.1111/j.1749-6632.1984.tb23417.x 6588812

[ref35] Giersch A. Wilquin H. Capa R. L. Delevoye-Turrell Y. N. (2013). Combined visual and motor disorganization in patients with schizophrenia. Front. Psychol. 4:620. doi: 10.3389/fpsyg.2013.00620, 24065934 PMC3776573

[ref36] Glahn D. C. Ragland J. D. Abramoff A. Barrett J. Laird A. R. Bearden C. E. . (2005). Beyond hypofrontality: a quantitative meta-analysis of functional neuroimaging studies of working memory in schizophrenia. Hum. Brain Mapp. 25, 60–69. doi: 10.1002/hbm.20138, 15846819 PMC6871703

[ref37] Grady C. (2012). Trends in neurocognitive aging. Nat. Rev. Neurosci. 13, 491–505. doi: 10.1038/NRN3256, 22714020 PMC3800175

[ref38] Grant D. A. Berg E. A. (1948). A behavioural analysis of degree of reinforcement and ease of shifting to new responses in a Weigl-type card-sorting problem. J. Exp. Psychol. 38, 404–411. doi: 10.1037/h0059831, 18874598

[ref39] Gunning-Dixon F. M. Raz N. (2000). The cognitive correlates of white matter abnormalities in normal aging: a quantitative review. Neuropsychology 14, 224–232. doi: 10.1037/0894-4105.14.2.224, 10791862

[ref40] Harlow J. M. (1868). Recovery from the passage of an iron bar through the head. Public Massachusetts Med Soc 2, 327–347.

[ref41] Hemsley D. R. (1976). Attention and information processing in schizophrenia. Br. J. Soc. Clin. Psychol. 15, 199–209. doi: 10.1111/j.2044-8260.1976.tb00025.x 938824

[ref42] Holm L. Karampela O. Ullén F. Madison G. (2017). Executive control and working memory are involved in sub-second repetitive motor timing. Exp. Brain Res. 235, 787–798. doi: 10.1007/s00221-016-4839-6, 27885405 PMC5315705

[ref43] Howes O. D. Bukala B. R. Beck K. (2023). Schizophrenia: from neurochemistry to circuits, symptoms and treatments. Nat. Rev. Neurol. 20, 22–35. doi: 10.1038/s41582-023-00904-0 38110704

[ref44] Huys R. Studenka B. Rheaume N. Zelaznik H. N. Jirsa V. (2008). Distinct timing mechanisms produce discrete and continuous movements. PLoS Comput. Biol. 4:e1000061. doi: 10.1371/journal.pcbi.1000061, 18437236 PMC2329590

[ref45] Huys R. Studenka B. E. Zelaznik H. N. Jirsa V. K. (2010). Distinct timing mechanisms are implicated in distinct circle drawing tasks. Neurosci. Lett. 472, 24–28. doi: 10.1016/j.neulet.2010.01.047, 20117170

[ref46] Infante V. H. P. Fehlberg M. Saikumar S. Drewing K. Meinke M. C. Bennewitz R. (2025). The role of skin hydration, skin deformability, and age in tactile friction and perception of materials. Sci. Rep. 15:9935. doi: 10.1038/s41598-025-95052-4 PMC11929835 40121378

[ref47] Jerde T. E. Soechting J. F. Flanders M. (2003). Coarticulation in fluent fingerspelling. J. Neurosci. Off. J. Soc. Neurosci. 23, 2383–2393. doi: 10.1523/JNEUROSCI.23-06-02383.2003, 12657698 PMC6742053

[ref48] Kelso J. A. S. (1995). Dynamic Patterns: The Self-Organization of Brain and Behavior. Cambridge, MA: MIT Press.

[ref49] Kennedy K. M. Raz N. (2009). Aging white matter and cognition: differential effects of regional variations in diffusion properties on memory, executive functions, and speed. Neuropsychologia 47, 916–927. doi: 10.1016/J.NEUROPSYCHOLOGIA.2009.01.001, 19166865 PMC2643310

[ref50] Koike S. Takizawa R. Nishimura Y. Kinou M. Kawasaki S. Kasai K. (2013). Reduced but broader prefrontal activity in patients with schizophrenia during n-back working memory tasks: a multi-channel near-infrared spectroscopy study. J. Psychiatr. Res. 47, 1240–1246. doi: 10.1016/j.jpsychires.2013.05.009, 23743135

[ref51] Koning J. P. F. Tenback D. E. Van Os J. Aleman A. Kahn R. S. Van Harten P. N. (2010). Dyskinesia and Parkinsonism in antipsychotic-naive patients with schizophrenia, first-degree relatives and healthy controls: a meta-analysis. Schizophr. Bull. 36, 723–731. doi: 10.1093/schbul/sbn146, 18990712 PMC2894597

[ref52] Koziol L. F. Lutz J. T. (2013). From movement to thought: the development of executive function. Appl. Neuropsychol. Child 2, 104–115. doi: 10.1080/21622965.2013.748386, 23848244

[ref53] Landelle C. Chancel M. Blanchard C. Guerraz M. Kavounoudias A. (2021). Contribution of muscle proprioception to limb movement perception and proprioceptive decline with ageing. Curr. Opin. Physio. 20, 180–185. doi: 10.1016/j.cophys.2021.01.016

[ref54] Leung H. T. Killcross A. S. Westbrook R. F. (2013). Error correction in latent inhibition and its disruption by opioid receptor blockade with naloxone. Neuropsychopharmacology 38, 2439–2445. doi: 10.1038/npp.2013.144, 23748224 PMC3799063

[ref55] Liu J. Riesch S. Tien J. Lipman T. Pinto-Martin J. O’Sullivan A. (2022). Screen media overuse and associated physical, cognitive, and emotional/behavioral outcomes in children and adolescents: an integrative review. J. Pediatr. Health Care 36, 99–109. doi: 10.1016/j.pedhc.2021.06.003, 34334279 PMC10029815

[ref56] Lu K. Nicholas J. M. Weston P. S. J. Stout J. C. O’Regan A. M. James S. N. . (2021). Visuomotor integration deficits are common to familial and sporadic preclinical Alzheimer’s disease. Brain Commun. 3:fcab003. doi: 10.1093/BRAINCOMMS/FCAB003 PMC7882207 33615219

[ref57] Luria A. R. (1966). Higher Cortical Functions in Man. New York, NY: Basic Books.

[ref58] Maeneja R. Rato J. Ferreira I. S. (2025). How is the digital age shaping young minds? A rapid systematic review of executive functions in children and adolescents with exposure to ICT. Children (Basel, Switzerland) 12:555. doi: 10.3390/CHILDREN12050555, 40426734 PMC12109849

[ref59] Maes P.-J. Wanderley M. M. Palmer C. (2015). The role of working memory in the temporal control of discrete and continuous movements. Exp. Brain Res. 233, 263–273. doi: 10.1007/s00221-014-4108-5, 25311387 PMC4290013

[ref60] Miyake A. Friedman N. P. (2012). The nature and Organization of Individual Differences in executive functions four general conclusions. Curr. Dir. Psychol. Sci. 21, 8–14. doi: 10.1177/0963721411429458, 22773897 PMC3388901

[ref61] Miyake A. Friedman N. P. Emerson M. J. Witzki A. H. Howerter A. Wager T. D. (2000). The unity and diversity of executive functions and their contributions to complex “frontal lobe” tasks: a latent variable analysis. Cogn. Psychol. 41, 49–100. doi: 10.1006/cogp.1999.0734, 10945922

[ref62] Muraven M. (2010). Building self-control strength: practicing self-control leads to improved self-control performance. J. Exp. Soc. Psychol. 46, 465–468. doi: 10.1016/j.jesp.2009.12.011, 20401323 PMC2855143

[ref63] Muraven M. Baumeister R. (2000). Self-regulation and depletion of limited resources: Does self-control resemble a muscle? Psychol. Bull. 126, 247–259. doi: 10.1037/0033-2909.126.2.247, 10748642

[ref64] Nardone R. Langthaler P. B. Schwenker K. Kunz A. B. Sebastianelli L. Saltuari L. . (2022). Visuomotor integration in early Alzheimer’s disease: a TMS study. J. Neurol. Sci. 434:120129. doi: 10.1016/j.jns.2021.120129, 34998240

[ref65] Norman W. Shallice T. (1986). “Attention to action,” in Consciousness and Self-Regulation: Advances in Research and Theory, (New York, NY: Plenum).

[ref66] O’Keefe J. Dostrovsky J. (1971). The hippocampus as a spatial map. Preliminary evidence from unit activity in the freely-moving rat. Brain Res. 34, 171–175. doi: 10.1016/0006-8993(71)90358-1, 5124915

[ref67] Ogden R. S. Salominaite E. Jones L. A. Fisk J. E. Montgomery C. (2011). The role of executive functions in human prospective interval timing. Acta Psychol. 137, 352–358. doi: 10.1016/j.actpsy.2011.04.004, 21561595

[ref68] Oppici L. Panchuk D. (2022). Specific and general transfer of perceptual-motor skills and learning between sports: a systematic review. Psychol. Sport Exerc. 59:102118. doi: 10.1016/J.PSYCHSPORT.2021.102118

[ref69] Peralta V. Campos M. S. De Jalón E. G. Cuesta M. J. (2010). Motor behavior abnormalities in drug-naïve patients with schizophrenia spectrum disorders. Mov Disord 25, 1068–1076. doi: 10.1002/MDS.23050, 20222137

[ref70] Planche V. Manjon J. V. Mansencal B. Lanuza E. Tourdias T. Catheline G. . (2022). Structural progression of Alzheimer’s disease over decades: the MRI staging scheme. Brain Commun 4, 1–10. doi: 10.1093/braincomms/fcac109, 35592489 PMC9113086

[ref71] Repp B. H. (2008). Perfect phase correction in synchronization with slow auditory sequences. J. Mot. Behav. 40, 363–367. doi: 10.3200/JMBR.40.5.363-367, 18782711

[ref72] Robertson S. D. Zelaznik H. N. Lantero D. A. Gadacz Bojczyk K. Spencer R. M. Doffin J. G. . (1999). Correlations for timing consistency among tapping and drawing tasks: evidence against a single timing process for motor control. J. Exp. Psychol. Hum. Percept. Perform. 25, 1316–1330. doi: 10.1037//0096-1523.25.5.1316, 10531665

[ref73] Roerdink M. Ophoff E. Peper C. E. Beek P. J. (2008). Visual and musculoskeletal underpinnings of anchoring in rhythmic visuo-motor tracking. Exp. Brain Res. 184, 143–156. doi: 10.1007/s00221-007-1085-y, 17724583 PMC2080346

[ref74] Roerdink M. Ridderikhoff A. Peper C. E. Beek P. J. (2012). Informational and neuromuscular contributions to anchoring in rhythmic wrist cycling. Ann. Biomed. Eng. 41, 1726–1739. doi: 10.1007/s10439-012-0680-7, 23099793 PMC3701797

[ref75] Rogers R. D. Monsell S. (1995). Costs of a predictible switch between simple cognitive tasks. J. Exp. Psychol. Gen. 124, 207–231. doi: 10.1037/0096-3445.124.2.207

[ref76] Salthouse T. A. (1996). General and specific speed mediation of adult age differences in memory. J. Gerontol. B Psychol. Sci. Soc. Sci. 51B, P30–P42. doi: 10.1093/GERONB/51B.1.P30, 8548516

[ref77] Salthouse T. A. (2000). Aging and measures of processing speed. Biol. Psychol. 54, 35–54. doi: 10.1016/S0301-0511(00)00052-1, 11035219

[ref78] Samain-Aupic L. Dione M. C. Ribot-Ciscar E. Ackerley R. Aimonetti J. M. (2024). Relations between tactile sensitivity of the finger, arm, and cheek skin over the lifespan showing decline only on the finger. Front. Aging Neurosci. 16:1387136. doi: 10.3389/FNAGI.2024.1387136/FULL PMC11250473 39015473

[ref79] Schmidt R. A. Lee T. D. (2011). Motor Control and Learning: A Behavioural Emphasis. Champaign, IL: Human Kinetics.

[ref80] Schmidt J. R. Liefooghe B. De Houwer J. (2020). An episodic model of task switching effects: erasing the homunculus from memory. J. Cogn. 3:22. doi: 10.5334/JOC.97, 32964181 PMC7485406

[ref81] Shallice T. (1982). Specific impairments of planning. Philos Transa Royal Soc London B Biol Sci 298, 199–209. doi: 10.1098/rstb.1982.0082, 6125971

[ref82] Shallice T. (1988). From Neuropsychology to Mental Structure. Cambridge: Cambridge University Press.

[ref83] Shallice T. Burgess P. W. (1991). Deficits in strategy application following frontal lobe damage in man. Brain 114, 727–741. doi: 10.1093/brain/114.2.727, 2043945

[ref84] Simpson A. Riggs K. (2007). Under what conditions do young children have difficulty inhibiting manual actions? Dev. Psychol. 43, 417–428. doi: 10.1037/0012-1649.43.2.417, 17352549

[ref85] Simpson A. Riggs K. J. Beck S. R. Gorniak S. L. Wu Y. Abbott D. . (2012). Refining the understanding of inhibitory processes: how response prepotency is created and overcome. Dev. Sci. 15, 62–73. doi: 10.1111/J.1467-7687.2011.01105.X, 22251293 PMC3405835

[ref86] Skedung L. El Rawadi C. Arvidsson M. Farcet C. Luengo G. S. Breton L. . (2018). Mechanisms of tactile sensory deterioration amongst the elderly. Sci. Rep. 8:5303. doi: 10.1038/s41598-018-23688-6, 29674633 PMC5908919

[ref87] Smith G. L. Large M. M. Kavanagh D. J. Karayanidis F. Barrett N. A. Michie P. T. . (1998). Further evidence for a deficit in switching attention in schizophrenia. J. Abnorm. Psychol. 107, 390–398. doi: 10.1037/0021-843X.107.3.390, 9715574

[ref88] Stadlbauer U. Langhans W. Meyer U. (2013). Administration of the Y2 receptor agonist PYY3-36 in mice induces multiple behavioral changes relevant to schizophrenia. Neuropsychopharmacology 38, 2446–2455. doi: 10.1038/npp.2013.146, 23748226 PMC3799064

[ref89] Stevens K. E. Zheng L. Abrams D. J. (2013). Intermittent versus continuous central administration of clozapine in DBA/2 mice, improvement in sensory inhibition deficits. Schizophr. Res. 149, 121–126. doi: 10.1016/j.schres.2013.06.007, 23863771

[ref90] Studenka B. Zelaznik H. N. (2008). The influence of dominant versus non-dominant hand on event and emergent motor timing. Hum. Mov. Sci. 27, 29–52. doi: 10.1016/j.humov.2007.08.004, 18191491

[ref91] Sülzenbrück S. Hegele M. Rinkenauer G. Heuer H. (2011). The death of handwriting: secondary effects of frequent computer use on basic motor skills. J. Mot. Behav. 43, 247–251. doi: 10.1080/00222895.2011.571727, 21598156

[ref92] Tippett W. J. Sergio L. E. (2006). Visuomotor integration is impaired in early stage Alzheimer’s disease. Brain Res. 1102, 92–102. doi: 10.1016/j.brainres.2006.04.049, 16797495

[ref93] Treisman M. (1963). Temporal discrimination and the indifference interval: implications for a model of the “internal clock”. Psychol Monograp 77, 1–31. doi: 10.1037/h0093864 5877542

[ref94] Turvey M. (1977). “Preliminaries to a theory of action with reference to vision,” in Perceiving, Acting and Knowing, ed. Bransford R. E. S. J.. (Hillsdale, NJ: Erlbaum) 211–265.

[ref95] Verheij S. Muilwijk D. Pel J. J. M. Van Der Cammen T. J. M. Mattace-Raso F. U. S. Van Der Steen J. (2012). Visuomotor impairment in early-stage Alzheimer’s disease: changes in relative timing of eye and hand movements. J Alzheimer’s Dis 30, 131–143. doi: 10.3233/JAD-2012-111883, 22377783

[ref96] Viau-Quesnel C. Fortin C. (2014). Bivalent task switching and memory load: similar costs on reaction times, different costs on concurrent timing. Can. J. Exp. Psychol. Rev. Can. Psychol. Exp. 68, 194–203. doi: 10.1037/cep0000017, 25383477

[ref97] Vorberg D. Wing A. (1996). “Modeling variability and dependence in timing,” in Handbook of Perception and Action, eds. Herbert H. Steven W. K. (Cambridge, MA: Academic Press).

[ref98] Weintraub S. Wicklund A. (2012). The neuropsychological profile of Alzheimer disease. Cold Spring Harb. Perspect. Med. 2:a006171. doi: 10.1101/cshperspect.a006171, 22474609 PMC3312395

[ref99] West R. L. (1996). An application of prefrontal cortex function theory to cognitive aging. Psychol. Bull. 120, 272–292. doi: 10.1037/0033-2909.120.2.272, 8831298

[ref100] Wing A. Kristofferson A. (1973a). Response delays and the timing of discrete motor responses. Percept. Psychophys. 14, 5–12. doi: 10.3758/bf03198607

[ref101] Wing A. Kristofferson A. (1973b). The timing of interresponse intervals. Percept. Psychophys. 13, 455–460. doi: 10.3758/BF03205802

[ref102] Winges S. A. Furuya S. Faber N. J. Flanders M. (2013). Patterns of muscle activity for digital coarticulation. J. Neurophysiol. 110, 230–242. doi: 10.1152/jn.00973.2012, 23596338 PMC3727042

[ref103] Yogev H. Hadar U. Gutman Y. Sirota P. (2003). Perseveration and over-switching in schizophrenia. Schizophr. Res. 61, 315–321. doi: 10.1016/S0920-9964(02)00323-7, 12729883

[ref104] Yogev H. Sirota P. Gutman Y. Hadar U. (2004). Latent inhibition and overswitching in schizophrenia. Schizophr. Bull. 30, 713–726. doi: 10.1093/oxfordjournals.schbul.a007125, 15954186

[ref105] Yordanova J. Kolev V. Hohnsbein J. Falkenstein M. (2004). Sensorimotor slowing with ageing is mediated by a functional dysregulation of motor-generation processes: evidence from high-resolution event-related potentials. Brain 127, 351–362. doi: 10.1093/brain/awh042 14607784

[ref106] Zakay D. Shub J. (1998). Concurrent duration production as a workload measure. Ergonomics 41, 1115–1128. doi: 10.1080/001401398186423, 9715671

[ref107] Zelaznik H. N. Spencer R. M. Doffin J. G. (2000). Temporal precision in tapping and circle drawing movements at preferred rates is not correlated: further evidence against timing as a general-purpose ability. J. Mot. Behav. 32, 193–199. doi: 10.1080/00222890009601370, 11005948

[ref108] Zelaznik H. N. Spencer R. Ivry R. B. (2002). Dissociation of explicit and implicit timing in repetitive tapping and drawing movements. J. Exp. Psychol. Hum. Percept. Perform. 28, 575–588. doi: 10.1037/0096-1523.28.3.575 12075889

[ref109] Zhou S. Seay M. Taxidis J. Golshani P. Buonomano D. (2023). Multiplexing working memory and time: encoding retrospective and prospective information in neural trajectories. Nat. Hum. Behav. 7, 1170–1184. doi: 10.1038/s41562-023-01592-y, 37081099 PMC10913811

[ref110] Zimmermann P. Fimm B. (2004). “A test battery for attentional performance,” in Applied Neuropsychology of Attention, (East Sussex: Psychology Press).

[ref111] Zimmermann P. Fimm B. (1994). Tests d’évaluation de l’attention (TEA)-Version 2.1. Herzogenrath: Psytest.

